# Opioids and Ocular Surface Pathology: A Literature Review of New Treatments Horizons

**DOI:** 10.3390/jcm11051424

**Published:** 2022-03-04

**Authors:** Celia García-López, Carmen Gómez-Huertas, José-María Sánchez-González, Davide Borroni, Marina Rodríguez-Calvo-de-Mora, Vito Romano, Rahul Rachwani-Anil, Juan-Francisco Ramos-López, Santiago Ortiz-Pérez, Carlos Rocha-de-Lossada

**Affiliations:** 1 Department of Ophthalmology, Hospital Universitario Virgen de las Nieves, 18014 Granada, Spain; cegarcilop@gmail.com (C.G.-L.); carmen_8871@hotmail.com (C.G.-H.); oftalmoramos@hotmail.com (J.-F.R.-L.); drsantiagoortiz@gmail.com (S.O.-P.); carlosrochadelossada5@gmail.com (C.R.-d.-L.); 2 Department of Physics of Condensed Matter, Optics Area, University of Seville, 41004 Seville, Spain; 3 Department of Doctoral Studies, Riga Stradins University, LV-1007 Riga, Latvia; info.borroni@gmail.com; 4 Cornea Research Unit, ADVALIA Vision, 20145 Milan, Italy; 5 Department of Ophthalmology, Hospital Regional Universitario de Málaga, 29010 Málaga, Spain; marocalmo@gmail.com; 6 Department of Ophthalmology (Qvision), Vithas Almería, 04120 Almería, Spain; 7 Department of Eye and Vision Science Ophthalmology, St Paul’s Eye Hospital, Liverpool L7 8XP, UK; vito.romano@gmail.com; 8 Institute of Life Course and Medical Sciences, University of Liverpool, Liverpool L69 3BX, UK; 9 Department of Medical and Surgical Specialties, Radiological Sciences, and Public Health, Ophthalmology Clinic, University of Brescia, 25121 Brescia, Italy; 10 Department of Ophthalmology, Hospital de Antequera, 29200 Málaga, Spain; rahul.medum@gmail.com; 11 Department of Surgery, Faculty of Medicine, University of Granada, 18010 Granada, Spain; 12 Department of Ophthalmology, Ceuta Medical Center, 51001 Ceuta, Spain

**Keywords:** cornea, opioids, wound healing, ocular surface, neuropathic corneal pain

## Abstract

This review discusses the role of opioids in the corneal surface and the different pathways and therapeutic methods of management. A literature review was performed using PubMed database. For the database search, the main searching words “opioid” and “topical opioid treatment” were used with the descriptors “cornea”, “ocular surface”, “neuropathic corneal pain”, “corneal sensitivity” and “naltrexone”; original scientific articles and reviews were included to achieve the purpose of the review. The endogenous opioid system has relevant functions in the organism, and in daily use, opioids are used as painkillers. However, these drugs may be employed for other indications as opioid pathways have a wide spectrum. The corneal surface for topical treatment is easily accessible, hence sparing the side effects of systemic opioids. Instillation of opioid antagonist substances, such as naltrexone, increases corneal healing rates and stimulates the division of corneal epithelium cells without deleterious effects. The natural modulation of endogenous opioids controls different forms of pain, including inflammatory and neuropathic pain, both in the ocular surface and in the central nervous system. There are diverse methods in controlling pain using opioids, especially in refractory forms. This review attempts to collect the literature about corneal surface and opioid pathways to provide an overview image and a possible direction of the news treatments.

## 1. Introduction

The endogenous opioid system is formed by a series of neuropeptides, namely: enkephalins, divided into leucine-enkephalins and methionine-enkephalins, endorphins and dynorphins. These neuropeptides exert their functions through the widely expressed opioid receptors in the central and peripheral neurons, and in the neuroendocrine, immune, and ectodermal cells [1]. These receptors belong to the family of G-protein coupled receptors that are divided into two groups. The classical receptors are mu (MORs, mainly expressed in the central nervous system (CNS), delta (DORs, expressed in the CNS and spinal cord), and kappa (KORs, expressed primarily in the spinal cord); and the non-classical receptors are the opioid growth factor receptor (OGFr) that are expressed in multiple targets organ [1,2,3]. The binding to classical and non-classical opioid receptors produces relevant biologic functions such as analgesia, hypoxia, limbic system modulation, neuroprotection, cardiovascular and respiration control, embryonic development, and cell division and growth [4].

The opioid system is also present in the eye. Endogenous opioids and their receptors are expressed in the mammalian retina [5,6]. Some of the classical opioid receptors, such as DORs [7] and MORs [8], have been found in retinal pigmented epithelial cells and in ganglion cells, respectively, with a neuroprotection role against ischemia [9]. Similarly, in the anterior ocular segment, KORs have been detected in the nonpigmented ciliary epithelium and trabecular meshwork cells of the anterior chamber with a role in intraocular pressure regulation [10].

Concerning the ocular surface, and especially in the cornea, the presence and the role of the endogenous opioid system owes a special importance. Epithelium and corneal terminal nerves possess a large number of receptors, including OGFr, MORs, and DORs, which establish an intricate relationship of mutual support [11,12,13,14]. The terminal nerves of the cornea, supplied by the ophthalmic division of the trigeminal nerves, are exclusively formed by C- and A-delta fibers with terminal receptors that sense pain, cold, mechanical and chemical stimuli, and tear production [15]. As with other peripheral nerve systems, corneal nerve fibers release endogenous met- and leu-enkephalins peptides in the basal form [13] and play a role in modulating acute and chronic pain [1].

Part of the normal immune system present in the ocular surface, such as lymphocytes, dendritic cells, and monocytes, release enkephalins and dynorphins [16]. These cells and native opioids, recruited especially in inflammatory situations, have demonstrated a function in analgesia and neuropathic pain [17,18,19]. In vitro studies, opioids have shown the ability to modulate immune responses, including cell proliferation, chemotaxis, cytotoxicity, cytokine synthesis and secretion, and chemokine receptor expression [11].

Due to the presence of opioids receptors on peripheral sensory nerve endings and immune cells in other accessible locations such as the skin, local opioid applications have been used for alleviating pain in patients with different inflammatory lesions, burns, arthritis, acute or chronic wounds [11].

However, the experience to modulate the opioid system in the ocular surface is more limited. The use of known exogenous opioids [20] and the pharmacology synthesis of new agonists, antagonists, or modulators of opioid receptors [21] holds the possibility for manipulation of these pathways and the finding of new targeted therapies for common ocular surface problems like delayed corneal wounds, dry eye disease, and neuropathic and chronic ocular pain. 

The aim of our review is to portray the evidence of the opioid therapeutic targets in the ocular surface, their clinical approach, and future treatments.

## 2. Materials and Methods

The research question is: which are the actual therapeutics options to use the opioid system in the pathology of ocular surface, especially as topical treatment?

### 2.1. Aims of the Research

- Search physiopathology information of the opioid system related to the ocular surface from the current evidence base.
- Explore the different possibilities of manipulation of the pathway and the therapeutic effect.
- Search the current experimental studies in mammals about this topic (independent of the phase of the study) and the results with a special interest in topical treatment.

### 2.2. Search Structure

For achieving the aims associated with the research question, a narrative literature search was performed in PubMed using the keywords: “opioid” AND “cornea” OR “ocular surface”, “topical opioid treatment” AND “cornea”, “neuropathic corneal pain” OR “corneal sensitivity” AND “opioid, naltrexone” AND “cornea”.

A total of 127 studies were identified for inclusion in the literature review, which covered the period of 2000 to October of 2021. A review of the obtained abstracts was undertaken and 85 articles were selected by the reviewer.

The references of the articles were cross-matched to achieve 28 relevant articles more to be included in the literature review. With the aim to collect the different forms to modulate the opioid system in the cornea, this article has been divided according to the main functions of the opioid system in the ocular surface.

## 3. Discussion

### 3.1. Division and Growth Factor Function

#### 3.1.1. OGF-OGFr Axis

Met-enkephalin, identified as an opioid growth factor (OGF), is an autocrine and paracrine peptide produced by the organism as a potent and non-specific tissue opioid [1]. The pathway formed by the OGF and its receptor (OGFr) serves as a tonically repressive axis that inhibits DNA synthesis and supposes a strong inhibitory signal for the growth and migration of the cells [22].

The suppression of the pathway with an antagonist opioid, especially naltrexone (NTX), achieves the effect of increased cell division and has been studied in the onset and progression of autoimmune diseases, cancer, and re-epithelization [23,24]. NTX is a long-acting opioid antagonist that acts on all opioid receptors, especially blocking the interaction between met-enkephalins and OGFr and MORs. The duration of the OGFr blockade determines the biotherapeutic response [25].

The intermittent blockade by low dosages of NTX produced depressed cell replication and a low and unique dosage of NTX reduced DNA system, generating a smaller body and brain in rats. In contrast, continuous blockage of the receptor with a repetitive application or sustained levels of NTX increased cell replication and generated a larger body and brain in rats [25,26].

#### 3.1.2. OGF-OGFr Axis in Cornea

Met enkephalins are produced by basal and suprabasal corneal epithelial cells, as well as limbal and conjunctival cells in normal conditions [27], and exert their actions in the OGFr receptors present in the cornea [14].

Endogenous opioids have a role in corneal homeostasis [28]. OGF is an inhibitory growth factor. The reepithelization of the human cornea is modulated by the opioid growth system [25,29].

Systemic and topical NTX in frequent dosage and chronic treatment accelerates normal homeostatic processes of the epithelium and increases proliferation of epithelial cells in experimental animals with a minimal effect on stem cell proliferation [30]. The rapid growth and cell migration do not result in cell harm, abnormal morphology of the epithelium neoplastic, or proliferative pathology [30,31,32].

Similarly, the overexpression of OGFr in corneal epithelium, using the transferred gene of OGFr DNA, delays epithelialization and the attenuation of OGFr accelerate it in a model corneal erosions in eye of rats [33].

#### 3.1.3. OGF-OGFr Axis in Diabetic Cornea

Diabetes type 1 and 2 produces corneal tissue complications such as decreased corneal epithelial turnover, less tear production, delayed corneal wounds repair, diminishes corneal surface sensitivity, and even generates neuropathy symptoms [34,35]. Enkephalin levels are elevated in the serum of diabetic type 1 humans and animals, and the levels seemed to be consistent with hyperglycemia and time of evolution [36,37].

The onset and magnitude of diabetes complications are associated with the elevations of OGF tissue and serum levels in diabetic rats [36,38,39,40]. These complications appear with more frequency and severity in female animal models, similarly associated with descended estradiol levels and elevated OGF levels [40].

In diabetic animal models, the corneal epithelium had elevated levels of OGF and OGFr. Because OGF is an inhibitory growth factor, the dysregulation of the pathway contributes to the delay’s re-epithelialization of corneal abrasions [38,41]. Experimental animals studies show how the continuous suppression of OGF receptor with topical NTX (2–3 times per day) or high systemic dosage of NTX are effective in the renewal of the ocular epithelium, normalized re-epithelization, and accelerating epithelial wound closure in diabetic animals though also in normal rats, mice and rabbits [30,42,43,44,45,46]. In addition, topical NTX was effective in attenuating corneal stroma neovascularization in the repair of the abraded cornea in diabetic rats. [47]. The early blockade of the OGF–OGFr axis with NTX could avoid the intensity and onset of complications of dysregulation of the axis in rats with diabetes type 1 [38].

### 3.2. Ocular Pain

The nociception system provides peripheral nerves with a framework of neural feedback to the central nervous system (CNS) in order to avoid or detect noxious stimuli. Pain disorders can be categorized into three groups: acute and nociceptive pain, neuropathic pain, and chronic pain. Nociceptive pain is produced by tissue damage secondary to noxious stimuli such as mechanical, surgical, or chemical stimulation. This pain becomes inflammatory pain when the stimulus persists, recruiting proinflammatory markers and activating responsive inflammatory cells. Meanwhile, neuropathic pain is caused by direct damage to sensory nerves [48].

The pathways for opioid analgesia in corneal pain involves multiple locations of action. The cornea sends afferent projections to the trigeminal dorsal horn of the medulla, more towards the rostral pole of the trigeminal nucleus caudalis than to the caudal pole. Thus, the trigeminal spinal nucleus is the initial place where they reach the trigeminal afferent fibers that encode noxious sensory information from the face area, including the eye [49]. Among the relevant pathways for central processing of nociceptive signals, thalamic nuclei and parabrachial nuclei are sites where dorsal horn neurons send ascending projections. The distribution of corneal nerve afferences to the rostral pole of trigeminal nucleus caudalis suggests that the nociceptive signals from the cornea are preferentially mediated by parabrachial-projecting neurons in and not by the ascending thalamic pathway [50].

#### 3.2.1. Nociception and Postoperative Pain

Pain sensation can be modulated by endogenous and exogenous opioids throughout its receptors MOR, DOR, and KOR [51,52], presented in neurons of the dorsal root ganglia (DRG), trigeminal ganglia, and in the terminals of peripheral sensory nerves [53].

The analgesic efficacy of opioids depends on the interaction with the different opioid receptors, specially MOR and DOR [53]. Exogenous opioids are classified as strong (e.g., morphine or oxycodone) or weak opioids (e.g., tramadol, codeine) based on their activity and affinity on the MORs [54]. In the opioid family, some substance, such as tramadol, has a dual mechanism of action, weak opioid receptor agonist of MOR and a weak inhibitor of the reuptake of noradrenaline and serotonin [55,56]. For the dual mechanism, tramadol can potentiate the descending of pain through non-opioid vias [56].

For efficacy in pain control, systemic oral opioids have been used frequently for the control of postoperative pain after ophthalmic surgery, especially in the refractive area [57,58]. However, side effects including nausea, constipation, paragogic hyperalgesia, respiratory depression, addiction, tolerance, and dependence have limited their use [59].

Opioid-sparing strategies have been used to minimize the undesirable effects, e.g., the combination of weak oral opioid (e.g., codeine, tramadol) and non-opioid drug (e.g., paracetamol or NSAIDs) of systemic opioids by facilitating the use of the lowest effective dose of opioids. In this regard, combining an oral opioid (such as codeine or tramadol) and a non-opioid (such as paracetamol or nonsteroidal anti-inflammatory drugs) with feely or fixed dosage allow the decrease the effective dose of opioids [60].

Several studies have been evaluating these agonist combinations. Codeine associated with acetaminophen is superior to placebo [61,62] and as effective and safe as oxycodone/acetaminophen [63] in the control of pain in photorefractive keratectomy.

Another strategy to avoid the systemic side effect is the change of administration via the topical administration of opioids can activate the peripheral opioids receptors located on afferent sensory nerve terminals in peripheral tissues. The treatment with exogenous opioids over the cornea limits the systemic opioid effects for the little systemic absorption of ones [64]. In spite of the long-term use of opioids can delay healing in cutaneous wounds [65], in the cornea, studies point out that topical opioids do not delay its re-epithelization [66,67].

In nociception pain models in animals, topical morphine presents conflicting results in its target of alleviating corneal pain, and not all dosage patterns or pain situations showed the same analgesic effects [68,69,70]. In a human model, 0.5% morphine drops every two hours in patients with photorefractive keratectomy showed control of pain without differences in epithelial rating or refractive outcomes [71].

Others incongruent results appear when topical fentanyl is used for controlling nociceptive pain [72,73]. Topical fentanyl treatment four times per day does not achieve alleviating the pain in patients with corneal erosions [73]. Other opioids such as tramadol, the opioid with a dual mechanism of action, have been studied on the corneal surface with enough topical tolerance in the ocular surface but without achieving an analgesic effect [74].

#### 3.2.2. Inflammatory Pain

Opioids have analgesic effects outside of the CNS. A large proportion of the analgesic effects produced by systemically administered opioids can be mediated by peripheral opioid receptors [11]. Painful inflammation of peripheral tissue (of varying duration) was studied as a regulatory stimulus of opioid receptors plasticity in adult sensory neurons. During a painful and inflammatory state, quantitative increments of MOR expression in the cornea and the ophthalmic branch of trigeminal ganglion appear [11,75,76]. In parallel, the nerve injury induces a local increase in met and leu enkephalins secretion by the terminal nerve fibers, thus attracting inflammatory cells and lymphocytes [77].

Thus, endogenous opioid peptides can control inflammatory pain and can reduce the release of proinflammatory molecules, showing an analgesic efficacy comparable with morphine [11,78]. However, one of the main obstacles to using endogenous opioids is their relative instability in vivo and its transitory effect due to the rapid degradation by neural endopeptidase neprylisin (NEP) and aminopeptidase N (APN) present in the eye surface [79]. A form to evade this metabolic problem is blocking these catabolic enzymes. Although the blockage of either enkephalinase or aminopeptidase do not result in significant analgesia in humans or animals, the simultaneous blockage of both peptidases achieve a potent analgesia [1,80].

Some substances, such as PL265, act as dual enkephalinase inhibitors (DENKI) of the catabolic enzymes NEP, APN, and LTA4 Hydrolase (LTA4H) and have shown antinociceptive and anti-inflammatory properties in the cornea of mice. The activation of corneal opioidergic receptors by elevated extracellular concentrations of enkephalins protected from their normal degradation could be an effective form to alleviate pain in several corneal injuries (toxic, traumatic, and inflammatory), making DENKIs a useful new class of topical analgesics [81]. Additionally, PL265 inhibits the extracellular matrix proteases and their degradation process, improving corneal wound healing [81].

This strategy against pain is based on the theoretical hyper-local effect of opioids. Enkephalins are physiologically released at needed areas, and DENKIs only permit the increase of the duration and quantity of them in these needed areas [1].

The undesirable side effects linked by exogenous administration of opioids have yet to be demonstrated with DENKIs [80].

However, the analgesic action of DENKIs may not be exclusive to the opioid pathway. Blockage of NEP protects from alterations and loss of corneal nerves due to avoidance of the degradation of other neuroprotector neurotransmitters associated with nerve regeneration [82].

According to elevated expression of MORs during inflammatory corneal pain, selective agonists of MOR such as DAMGO (D-Ala2, N-Me-Phe4Gly5-ol) may be useful in its control. The effect of MOR agonists may control the painful excitability of the corneal fibers. The topical application of DAMGO reversed the elevated activity of the ciliary nerve and the responsiveness of corneal nociceptors in a model of inflammatory pain in mice without exacerbated excitability of the fibers or hypersensitivity [8]. Likewise, the infiltration of inflammatory cells is able to modulate the activity of the corneal nerves, with the sensitization of nociceptors and reducing the responsiveness of cold thermoreceptors [83]. 

The current experimenting studies report DENKIs as a useful new class of topical analgesics, including the eye surface. The possibility to extend the average life of endogenous opioids provides a novel form to alleviate the pain with the great benefit of increasing a natural pathway to control the pain and the inflammatory states.

#### 3.2.3. Neuropathic Pain

According to the International Association for the Study of Pain, neuropathic pain is defined as “pain that arises as a direct consequence of a lesion or diseases affecting the somatosensory system” [84]. Hence, neuropathic corneal pain (NCP) could be defined as the outcome of the trauma to the corneal somatosensory system [84,85].

Persistent inflammation linked to tissue and peripheral axonal nerve damage can cause a chronic pain situation. This process results in peripheral sensitization; an increased sensitivity of peripheral nerves with an intensifying peripheral pain signaling. Over time, persistent stimuli may derive central sensitization, with changes in the central nervous system, conforming to a type of pain disconnected from peripheral signs [85,86]. Pain symptoms such as spontaneous pain, allodynia (pain without noxious stimuli), or hyperalgesia (exaggerated pain answer) can be indicative of sensitization [84].

The evidence of central sensitization or central component of the pain can be revealed with a lack of complete resolution of the pain with topical anesthesia [87]. In addition, photoallodynia or light sensitivity has been associated with centralized trigeminal pain [88].

NCP has multiple underlying mechanisms: peripheral triggers such as ocular surgery or ophthalmic herpes zoster; or systemic causes, such as diabetes, and other small-fiber polyneuropathy or fibromyalgia [86]. Moreover, more severe symptoms of neuropathic ocular pain are associated with comorbid psychiatric disease, such as anxiety or depression [89]. In addition, some authors highlight the similarities between dry eye and neuropathic pain [85,90,91].

The current therapy for NCP is not satisfactory [92]. Very often, traditional therapies such as topical corticosteroids, nonsteroidal anti-inflammatory drugs, topical cyclosporine, autologous serum eye drops, or intense lubrication do not improve NCP [93]. Refractory peripheral symptoms and central neuropathic pain are treated with systemic medical therapy with neuroleptics such as gabapentin [94] and pregabalin [95], with non-uniform answers of surgical therapies such as corneal neurotization [96] or intranasal neurostimulation [97].

The etiology of neuropathic and chronic pain is associated with the opioid system. In a model of corneal surface injury in mice with change and damage of the corneal nerves, the corneal injury-induced central and peripheral opioid receptor-dependent showed latent sensitization [98]. Latent sensitization is a long-lasting model of chronic pain in which hyperalgesia is continuously suppressed by the action of opioid receptors. This model can be demonstrated by the apparition of hyperalgesia or the induction of mechanical allodynia by opioid antagonists unmasking the sensitization. This model suggests that opioid receptors, especially MORs presented in nociceptive afferents, mediate an ongoing suppression of hyperalgesia to produce remission from chronic pain [99].

Oral opioids have been used in neuropathic pain in dry-eye patients with a high scale analgesia grade, and centralized pain has also been treated with opioids [86,91,100].

Within the opioids and the study of the etiology of neuropathic pain, tapentadol is an opioid with MOR agonist activity and noradrenaline reuptake inhibition with a central pain action [101]. Tapentadol potentiates the descending pain inhibition in patients with chronic diabetic polyneuropathy [102] and in fibromyalgia patients with a normal corneal nerve fiber state (CNFS), although it does not achieve analgesic effect in patients with abnormal CNFS [103].

Another form to modulate the endogenous opioid system is the increase of its release. The opioid rebound effect is the upregulation of opioid signaling with the rise of endogenous opioid production by the transient opioid receptor blockade [104]. Low-dose naltrexone (LDN) in doses of 1 to 5 mg involves this transient opioid receptor blockade, MOR and DOR, targeting analgesia through increasing endogenous endorphins. Moreover, LDN acts as an antagonist on toll-like receptor 4 in glial cells, suppressing microglial activation and, thereby, reducing the inflammatory response and neurotoxicity [104,105,106]. Following this effect, the efficacy of LDN has been used in diseases with chronic neuropathic pain such as fibromyalgia, Crohn’s disease, or refractory diabetic neuropathy [107,108].

LDN may ameliorate NCP in patients with central components of pain who had a refractory answer to other treatments. In this line, 4.5 mg of oral LDN for four weeks was used in refractory NCP patients with a centralized pain component with significant results in alleviating pain with good tolerability [105]. The main side effects linked to LDN are vivid dreams, headaches, and insomnia [106].

According to these results, preclinical reports in animals demonstrated that continuous suppressions of receptors with NTX applied topically resulted in reversal of dry eye and re-establishing the corneal sensitivity in diabetic animals without effect in normal ones [109,110]. Additionally, LDN (1–5 mg/daily) has been effective and well-tolerated for alleviating refractory neuropathic pain in diabetic patients [108,111].

The sustained hyperglycemia in diabetes produces the loss of corneal nerve fiber leading to decreased innervation to the cornea and sensitivity. The decreased corneal sensitivity result in dry eye, lower tear production, and even neuropathic pain [112]

However, NTX may disrupt this endogenous opioid–receptor interaction and restore tear production and corneal sensitivity in both type 1 and type 2 of diabetes, although the underlying mechanism is unclear [32,45].

Regarding the link between NTX and levels of OGF, serum levels of OGF were normal for rats receiving systemic NTX, and OGF tissue levels were normal for type 1 diabetic rats receiving twice-daily topical NTX, while OGFr levels remained elevated. These data support the role of OGF–OGFr binding in maintaining the onset of ocular surface complications and suggest the effect of NTX therapy may be useful in pre-diabetic and early diabetic states to prevent dry eye and aberrant corneal surface sensitivity [38].

**Figure 1:**
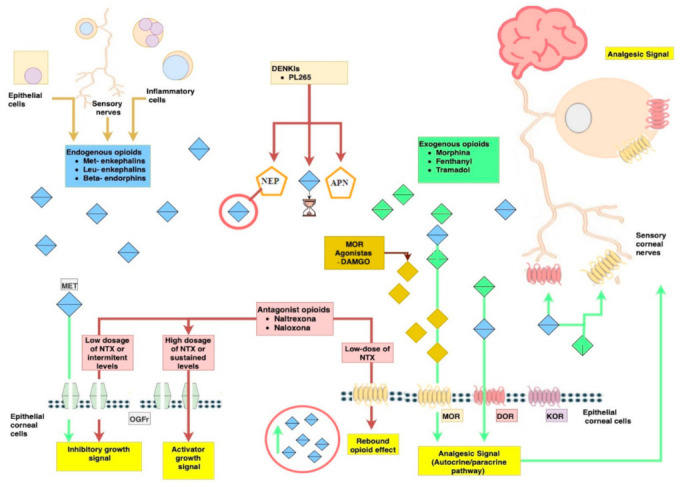
Endogenous opioids have different affinities from the opioid’s receptors (MORs, DORs, KORs, and OGFr) distributed by epithelial cells, endings terminal nerves, and trigeminal ganglion. In the synaptic space and in the cornea surface catabolic enzymes (NEP and APN) control the elimination and average life of endogenous opioids. There are multiples forms to control the pathway: the introduction to exogenous opioids (green rhomboids), the specific agonist of different opioids receptors, the use of antagonist of the via (Naltrexone) and his different dosage and levels, and the use of inhibitors of catabolic enzymes to achieve an increase of average life of natural enkephalins.

Based on animal experimentation evidence, topical administration of NTX might be more effective than systemic administration in hindering these abnormalities and future studies point out in that direction [105]. Focussing on this goal, pharmacology studies investigate the best form to get a prolonged release of NTX on the ocular surface and improve corneal penetration [113,114,115,116]. A recent small phase 1 study in healthy human volunteers reported tolerability to escalating doses of topical NTX (1–4 eye drops at dosages up to 50 μM) was completed with success [117] (Figure 1).

## Data Availability

The data presented in this study are available on request from the corresponding author.

## Abbreviations

APN	aminopeptidase N
DAMGO	D-Ala2, N-Me-Phe4Gly5-ol
DENKIs	dual enkephalinase inhibitors
DOR	delta opioid receptor
KOR	kappa opioid receptor
LDN	low-dose naltrexone
MOR	mu opioid receptor
NCP	neuropathic corneal pain
NEP	neural endopeptidase neprylisin
NTX	naltrexone
OGF	opioid growth factor
OGFr	opioid growth factor receptor
